# Supplementary material for: Vacuolar-type H+-ATPase-mediated extra-organellar buffering resolves mitochondrial dysfunction
Source: Nat Commun. 2025 Dec 3;17:67. doi: 10.1038/s41467-025-66656-1 (PMC12769673; doi:10.1038/s41467-025-66656-1)
Supplement: Supplementary file 1 — Supplementary Information [file 41467_2025_66656_MOESM1_ESM.pdf]

Supplementary Information

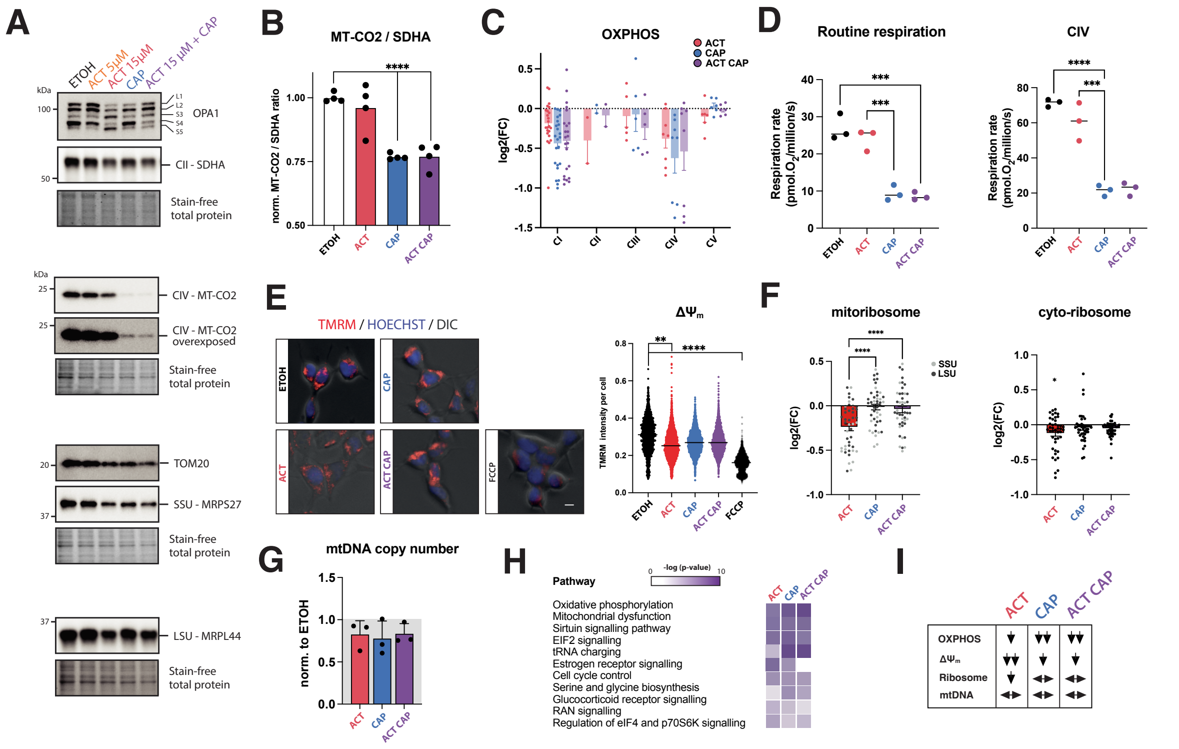

Figure S1 (related to Figure 1). *Characterization and functional analysis of the screen conditions.* (**A**) Immunoblotting of the screen conditions. Stress-induced OPA1 fragmentation, mitochondrial-nuclear imbalance (MT-CO2/SDHA), mitochondrial outer membrane protein (TOM20), mitochondrial ribosome stability (SSU: MRPS27; LSU: MRPL44). Loading control: Stain-free total protein.
(**B**) Mitochondrial-nuclear imbalance (MT-CO2/SDHA); label-free quantitative proteomics. Data represents the mean ratio ±SD of biological quadruplicates (dots), analyzed using a 1-way ANOVA.
(**C**) Steady-state abundance of OXPHOS subunits (CI-V). Data represents the means of biological quadruplicates for identified OXPHOS subunits relative to ETOH (dots).
(**D**) Oxygen consumption rates in the screen conditions; biological triplicates. Data represents the mean ±SD of three biological replicates (dots), 1-way ANOVA.
(**E**) Live-cell microscopy of $\Delta\Psi_m$ assessed via TMRM fluorescence in screen conditions and quantification; Positive control: $\Delta\Psi_m$-uncoupler FCCP. DNA stained with Hoechst. Scale bar: 5 μm. Data represents the mean with individual cells (dots) from representative staining (n>100), 1-way ANOVA.
(**F**) Expression level of detected mitoribosomal protein (MRP) and cytoplasmic ribosomal subunits relative to EtOH; label-free quantitative proteomics. Data represents the means of biological quadruplicates for identified MRP and cytoribosome subunits relative to ETOH (dots).
(**G**) Mitochondrial DNA (mtDNA) copy number in the screen conditions; quantitative PCR (qPCR). Data represents the mean ±SD of three independent experiments (dots).
(**H**) Pathway analysis of screen conditions; label-free quantitative proteomics. Top11 altered pathways.
(**I**) Differential effect of mitoribosomal inhibitors on mitochondrial parameters.

All treatments were performed in HEK293 cells for 15 days. Bars indicate the means of measurements with cells or independent experiments as dots. Statistical significance was performed using 1-way ANOVA, *p≤0.05, **p≤0.01. ***p≤0.001, ***p≤0.0001. Abbreviations: ACT = actinonin; CAP = chloramphenicol; FCCP = Carbonyl cyanide- p-trifluoromethoxyphenylhydrazone; LFQ = Label-free quantitation; LSU = large subunit mitoribosome; SSU = small subunit mitoribosome; TMRM = Tetramethylrhodamine methyl ester.

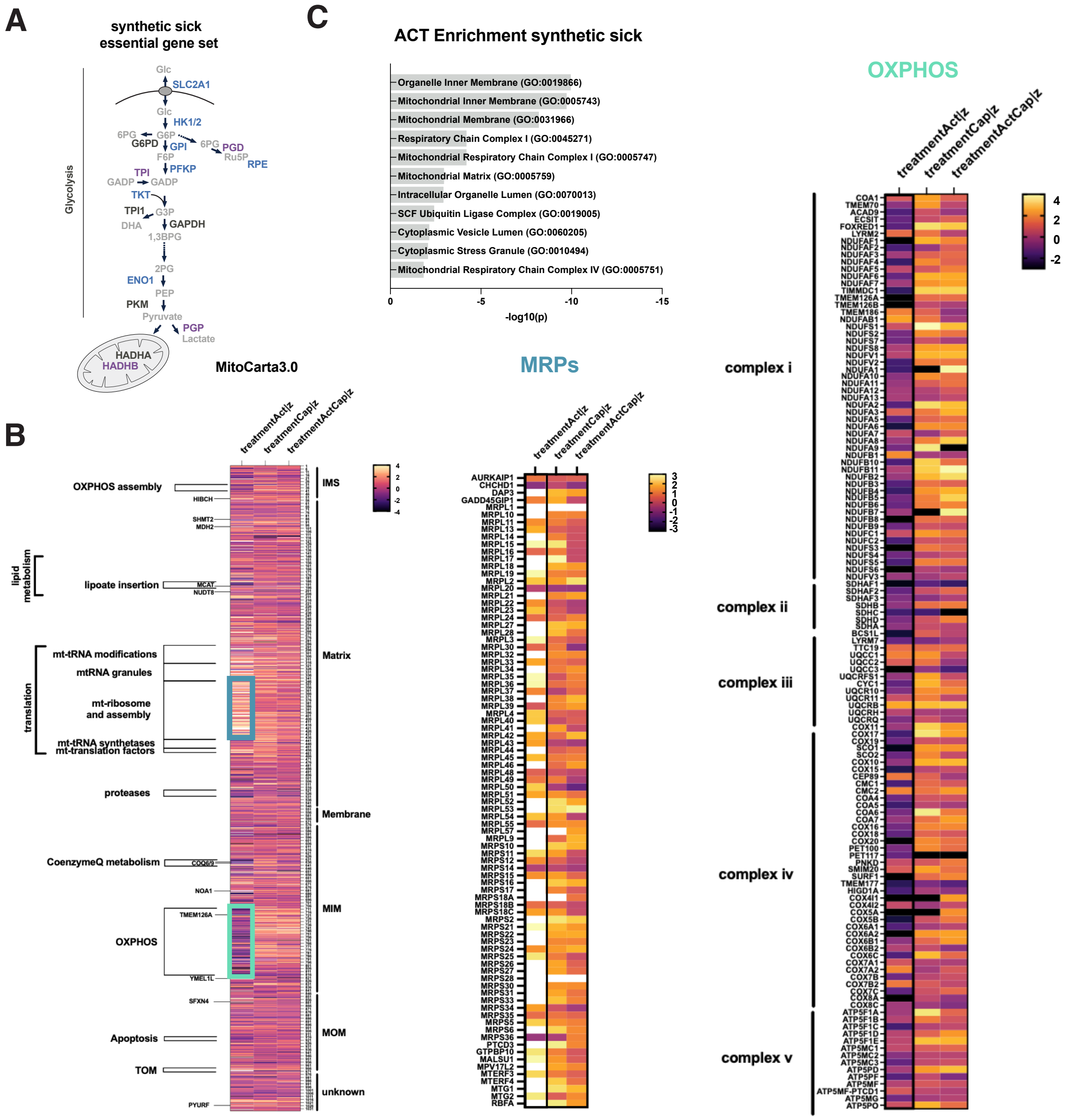

Figure S2 (related to Figure 1). *Compendium of mitochondrial mistranslation suppressors and synthetic sick/lethal genes.*

(**A**) Essential synthetic sick genes set in CAP (blue) and ACT CAP (unique to ACT CAP in purple).

(**B**) Heatmap of mitochondrial proteome (MitoCarta3.0) of suppressing and synthetic sick genes in mis- and nontranslation. OXPHOS and mitoribosome clusters are indicated and enlarged.

(**C**) Pathway analysis of synthetic sick genes in mitochondrial mistranslation (Z-score difference ≥ 3, ACT vs ACTCAP).

Abbreviations: ACT = actinonin; CAP = chloramphenicol; IMS = Intermembrane space; MIM = mitochondrial inner membrane; MOM = mitochondrial outer membrane; MRP – mitochondrial ribosomal proteins; TOM = translocase of outer membrane.

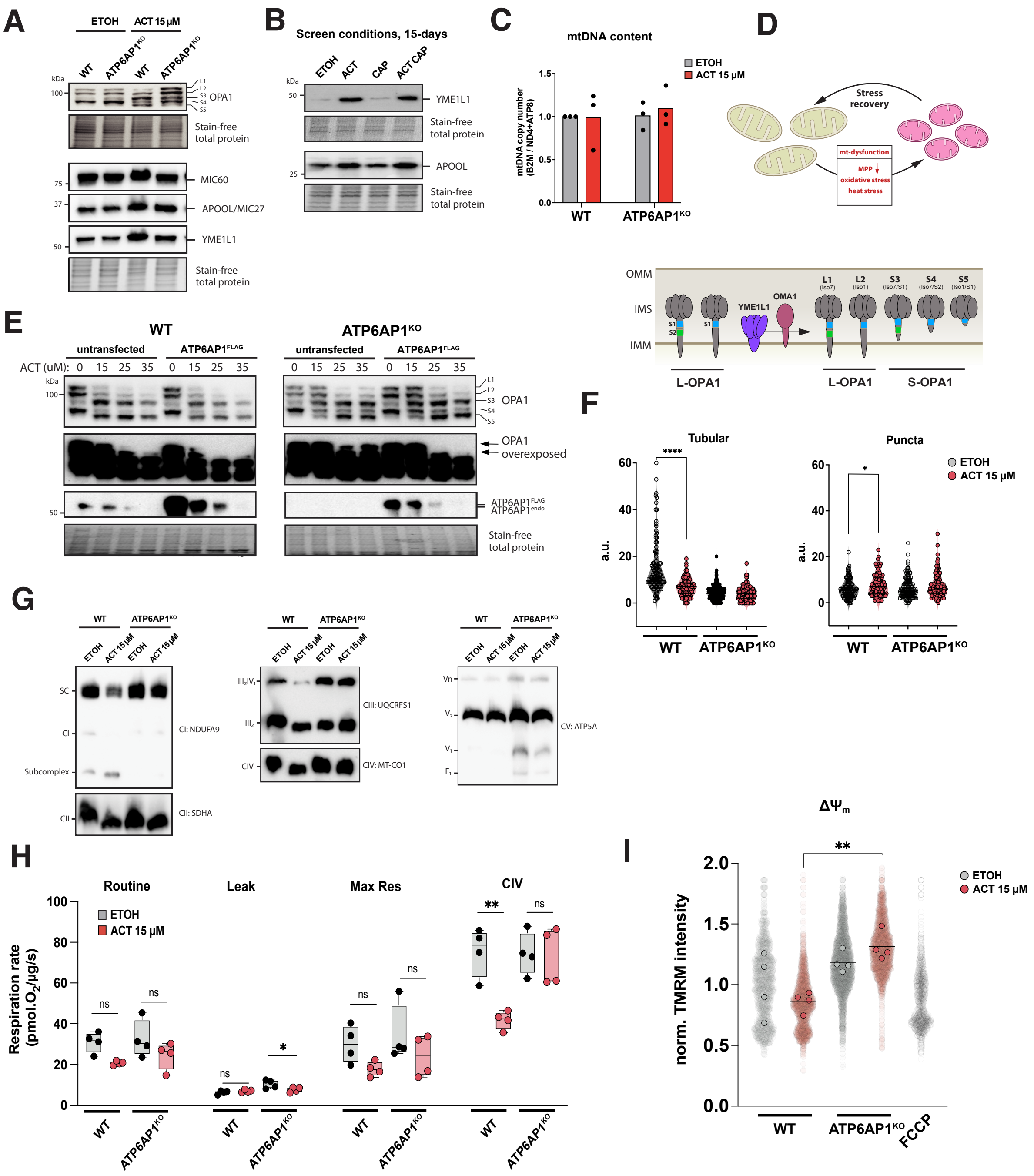

Figure S3 (related to Figure 2). *Characterization of loss of v-ATPase subunit ATP6AP1 in HEK293.* (**A**) Immunoblot of OPA1 processing and cristae organization markers; subunits MIC60/Mitofilin and APOOL/MIC27 of the inner membrane complex MICOS and OPA1-processing peptidase YME1L1 in ATP6AP1^{KO}.

(**B**) Immunoblot of cristae organization markers; subunits APOOL/MIC27 of the inner membrane complex MICOS and OPA1-processing peptidase YME1L1 in the screen conditions.

(**C**) qPCR to assess mtDNA) copy number in WT and ATP6AP1^{KO}. Data represents the mean ±SD of three independent experiments (dots), analyzed using a 1-way ANOVA.

(**D**) Schematic of OPA1-processing via YME1L1 and OMA1 and reversal of mitochondrial fragmentation.

(**E**) Complementation with WT FLAG-tagged ATP6AP1 in WT (left panel) and ATP6AP1^{KO} (right panel).

(**F**) Quantification of mitochondrial fragmentation status from Figure 3F. Violin plot from individual cells (dots), analyzed using a 2-way ANOVA.

(**G**) Native PAGE to assess OXPHOS complex assembly in WT and ATP6AP1^{KO}. Correct detection inferred from the expected sizes of endogenous OXPHOS complexes.

(**H**) Oxygen consumption in WT and ATP6AP1^{KO}. Data represents the mean ±SD of four independent experiments (dots), analyzed using a 2-way ANOVA.

(**I**) Quantification of TMRM signal intensity from Figure 3G; mean with each dot representing a single cell (n > 100). Positive control: FCCP. Data represents the mean of four independent experiments (dots), analyzed using a 2-way ANOVA.

All treatments performed in HEK293 cells for 3 days if not indicated otherwise. Bars indicate mean of measurements with dots representing independent experiments if not indicated otherwise. Statistical significance was performed using 1- or 2-way ANOVA, n.s. non-significant, *p≤0.05, **p≤0.01. ***p≤0.001, ***p≤0.0001. Abbreviations: ACT = actinonin; a.u. = arbitrary units; CAP= chloramphenicol; FCCP = carbonyl cyanide- p-trifluoromethoxyphenylhydrazone a.u. = arbitrary units.

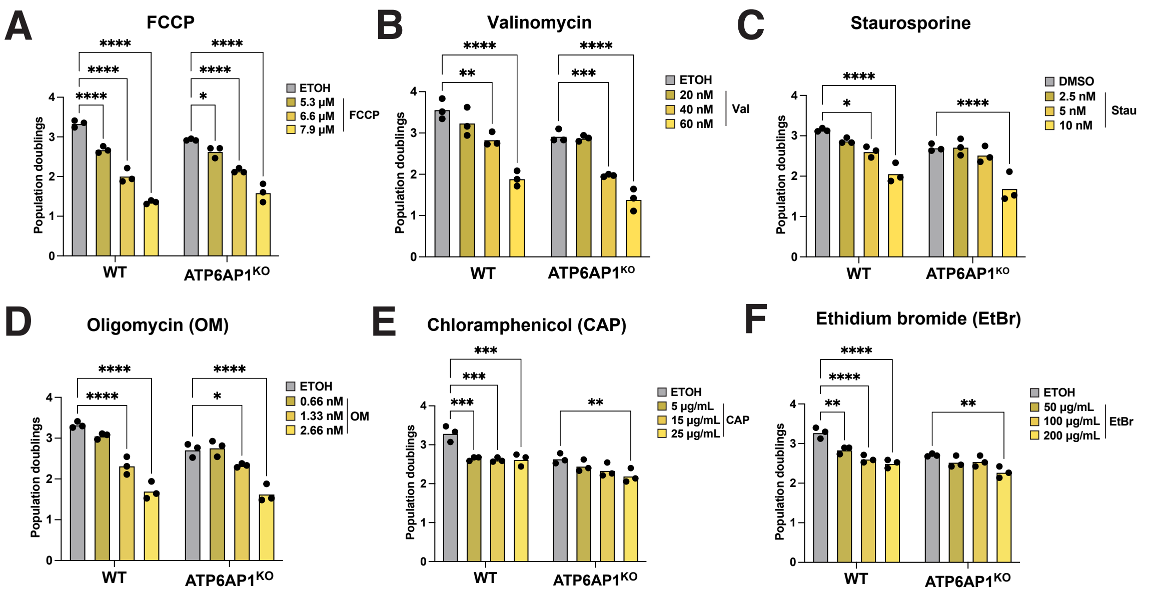

Figure S4 (related to Figure 2). *Characterization of sensitivity to various pharmacological inhibitors in ATP6AP1^{KO} in HEK293.*

(A) FCCP-induced growth inhibition in WT and ATP6AP1^{KO} cells.
(B) Valinomycin (Val)–induced growth inhibition in WT and ATP6AP1^{KO} cells.
(C) Staurosporine (Stau)–induced growth inhibition in WT and ATP6AP1^{KO} cells.
(D) Oligomycin (OM)–induced growth inhibition in WT and ATP6AP1^{KO} cells.
(E) Chloramphenicol (CAP)–induced growth inhibition in WT and ATP6AP1^{KO} cells.
(F) Ethidium bromide (EtBr)–induced growth inhibition in WT and ATP6AP1^{KO} cells.

All treatments were performed in HEK293 cells for 3 days if not indicated otherwise. Data represents the mean ±SD of three independent experiments (dots), 2-way ANOVA with *p≤0.05, **p≤0.01. ***p≤0.001, ****p≤0.0001.

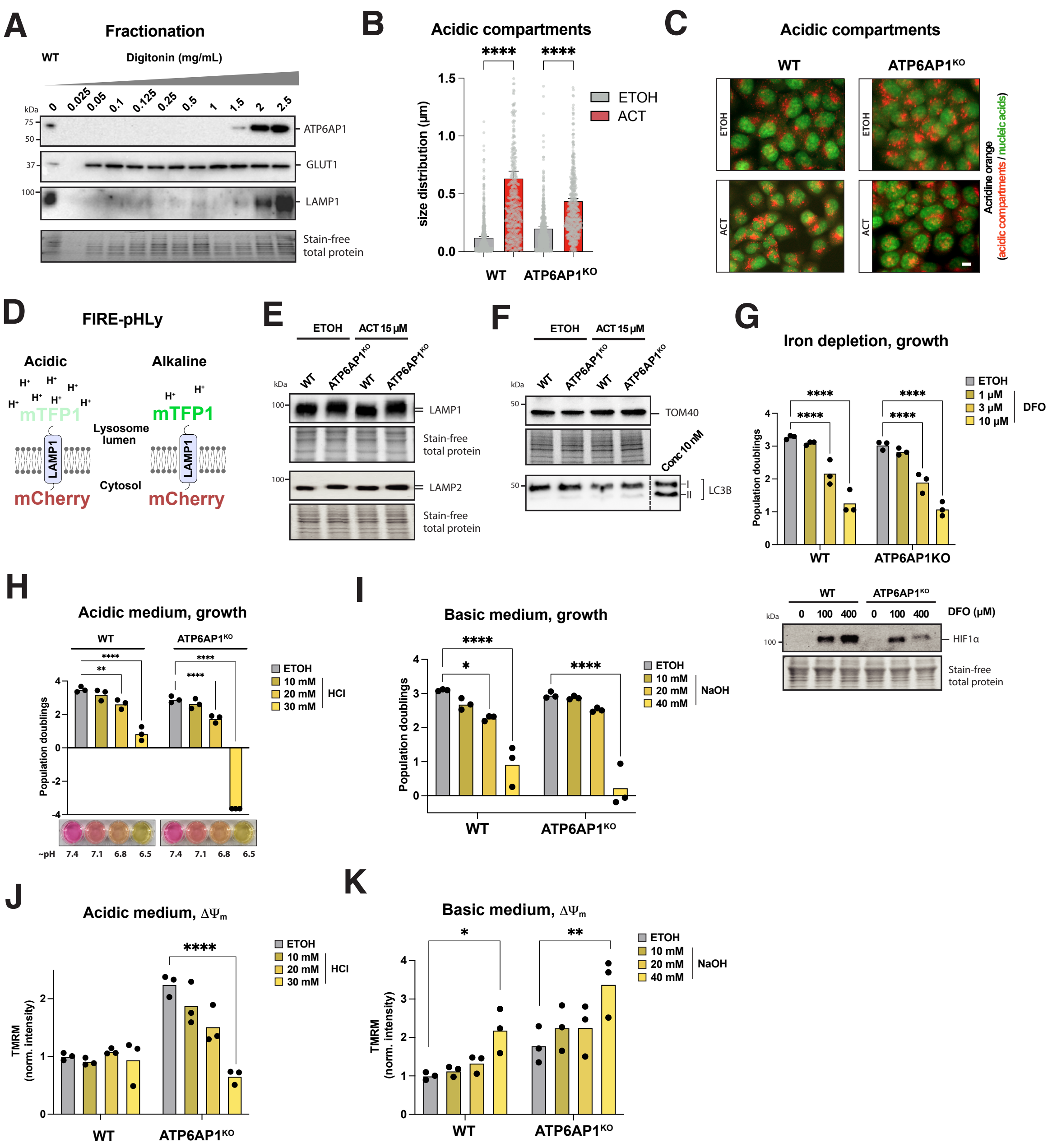

Figure S5 (related to Figure 3). *ATP6AP1 is associated with lysosomal v-ATPase and its loss increases the sensitivity to extracellular pH alterations.*

(**A**) Immunoblot of fractions from digitonin-titration of WT cells.

(**B**) Quantification of the acidic compartment size distribution in Figure 3E. Data represents the mean ±SEM with vesicles (dots) from representative staining (> 20 cells), 2-way ANOVA.

(**C**) Staining of acidic compartments by acridine orange.

(**D**) Working principle of the Fire-pHLY lysosomal biosensor.

(**E**) Immunoblotting against LAMP1, LAMP2.

(**F**) Immunoblotting against TOM40 and LC3B. Control for lipoylated LC3B is 1 h treatment of WT with 10nM concanamycin A.

(**G**) Growth phenotypes of WT and ATP6AP1^{KO} treated with the iron-chelator deferoxamine (DFO) (upper panel) and immunoblot of Hif1α with and without DFO (lower panel).

(**H**) Growth phenotypes of WT and ATP6AP1^{KO} with increasing extracellular acidification. Data represents the mean ±SD of three independent experiments (dots), 2-way ANOVA.

(**I**) Growth phenotypes of WT and ATP6AP1^{KO} with increasing extracellular alkalinization.

(**J**) $\Delta\Psi_m$ assessed via TMRM fluorescence intensity with increasing extracellular acidification in WT and ATP6AP1^{KO}.

(**K**) $\Delta\Psi_m$ assessed via TMRM fluorescence intensity with increasing extracellular acidification in WT and ATP6AP1^{KO}.

All treatments were performed in HEK293 cells for 3 days if not indicated otherwise. Data represents the mean ±SD of three independent experiments (dots), 2-way ANOVA (G-K); *p≤0.05, **p≤0.01. ***p≤0.001, ****p≤0.0001.

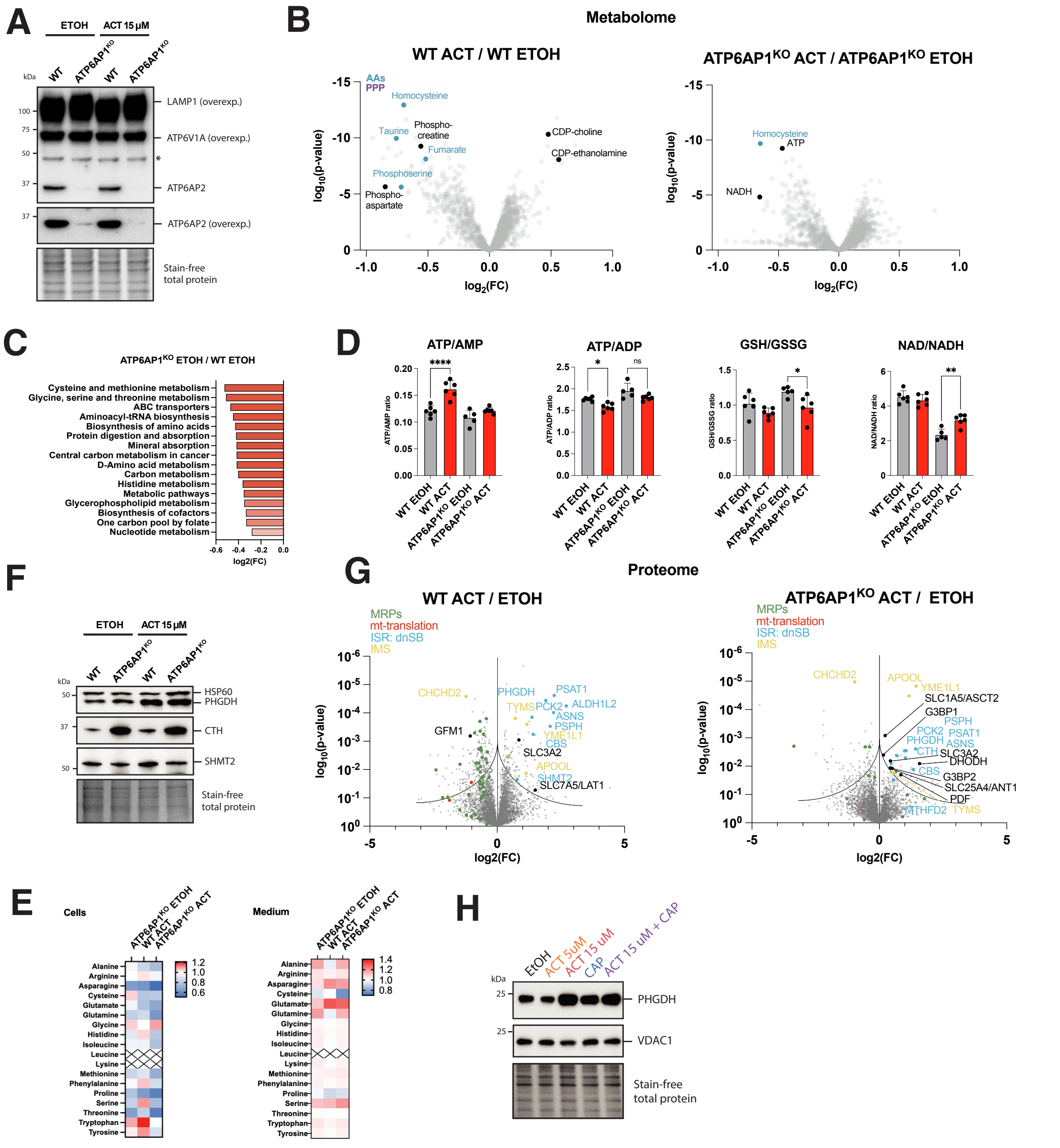

Figure S6 (related to Figure 4). *Proteomic and metabolomic alterations in v-ATPase subunit knockout of ATP6AP1 in HEK293.*

(**A**) Immunoblotting against LAMP1, ATP6V1A and ATP6AP2. Loading control: stain-free total protein. Asterisk indicates an unspecific band.

(**B**) Volcano plot of WT and ATP6AP1^{KO}; untargeted metabolomics; each dot represents the mean of biological sextuplicates; Student`s t-test.

(**C**) Altered metabolic pathways in WT and ATP6AP1^{KO}, all pathways p<0.05.

(**D**) Key metabolic ratios in WT and ATP6AP1^{KO}. Untargeted metabolomics; mean of biological sextuplicate normalized to WT or untreated control. Mean with ±SD. 2-way Anova.

(**E**) Heatmap of average amino acid level normalized to untreated control in cells and media from ATP6AP1^{KO}; untargeted metabolomics, mean of biological sextuplicate.
(**F**) Immunoblot steady state of metabolic stress enzymes PHGDH, HSP60, CTH and SMHT2.
(**G**) Volcano plot of WT and ATP6AP1^{KO}; label-free quantitative proteomics, each dot represents mean of quadruplicate measurements; Student`s t-test.
(**H**) Immunoblot of integrated stress response protein PHGDH and VDAC1 in conditions of the screen.

All treatments were performed in HEK293 cells for 3 days. Statistical significance was performed using 2-way ANOVA, **p≤0.05, **p≤0.01. ***p≤0.001, ****p≤0.0001.

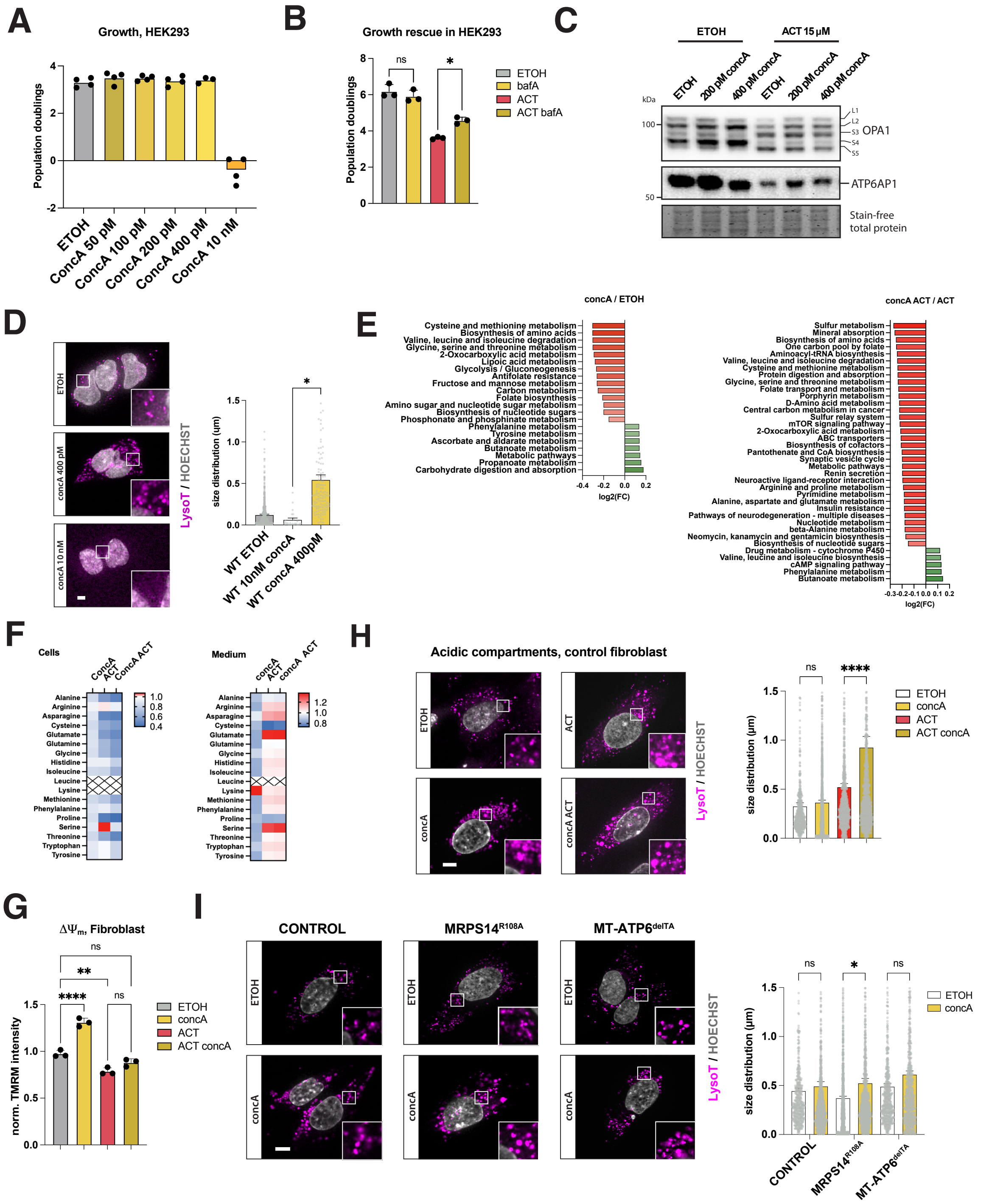

Figure S7 (related to Figure 5). **v-ATPase inhibition, growth adaptation, acidic vesicle quantification, and metabolic comparisons.**

(**A**) Growth phenotypes with increasing concentrations of concA. HEK293, 3 days treatment. Data represents the mean ±SD of three independent experiments (dots), 2-way ANOVA.

(**B**) Growth phenotypes with concomitant pharmacological v-ATPase inhibition using bafA under ACT treatment. HEK293, 6 days treatment. Data represents the mean ±SD of three independent experiments (dots), 2-way ANOVA.

(**C**) Immunoblot of OPA1-processing with concomitant pharmacological v-ATPase inhibition by increasing concA concentrations upon ACT treatment. HEK293, 3 days treatment.

(**D**) Quantification of acidic compartments with low (400pM) and high (10nM) concA; Lysotracker DND-99 fixed. DNA stained with Hoechst. Scale bar: 2 µm. Data represents the mean ±SEM with vesicles (dots) from representative staining (> 20 cells), 2-way ANOVA.

(**E**) Pathway analysis of 400 pM concA-treatment with and without ACT; untargeted metabolomics; HEK293, 3 days treatment. P-value cut-off <0.05; right-tailed Fisher's exact test.
(**F**) Heatmap of average amino acid level normalized to untreated control in WT cells and media from concA +/- ACT; untargeted metabolomics, mean of biological sextuplicates; 3-days treatment.
(**G**) $\Delta\Psi_m$ assessed via TMRM fluorescence intensity with increasing concA concentrations in fibroblasts derived from healthy control. Positive control: FCCP. Data represents the mean ±SD of three independent experiments (dots), 2-way ANOVA
(**H**) Quantification of acidic compartments in fibroblasts derived from healthy control. Lysotracker DND-99 fixed; Fibroblasts, 3 days treatment. DNA stained with Hoechst; Scale bar: 2 µm. Data represents the mean ±SEM with vesicles (dots) from representative staining (> 20 cells), 2-way ANOVA.
(**I**) Quantification of acidic compartments for healthy control- and patient-derived fibroblasts. Lysotracker DND-99 fixed; 3 days treatment. DNA stained with Hoechst; Scale bar: 2 µm. Data represents the mean ±SEM with vesicles (dots) from representative staining (> 20 cells), 2-way ANOVA.

All treatments performed in HEK293 or fibroblasts cells for 3 days. Bars indicate mean of measurements with vesicles or independent experiments as dots. Statistical significance of 2-way ANOVA ns. non-significant, *p≤0.05, **p≤0.01. ***p≤0.001, ****p≤0.0001.

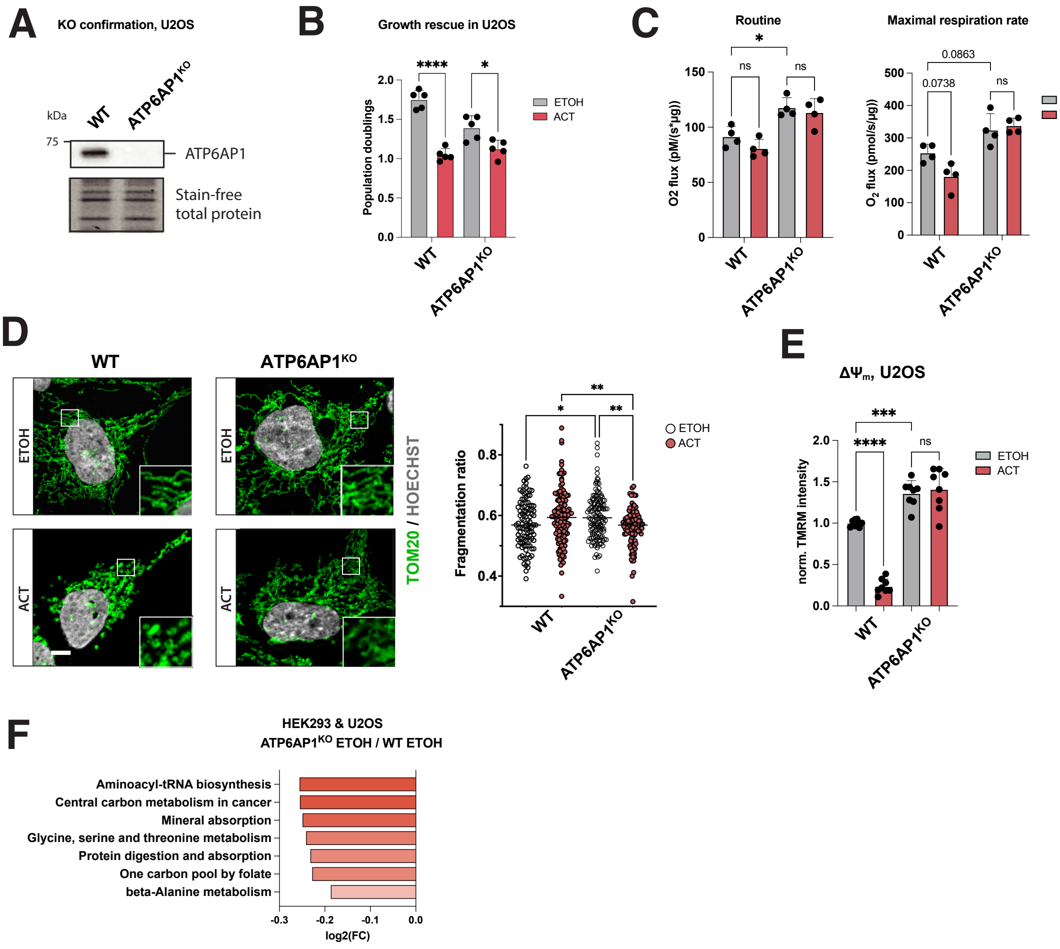

Figure S8 (related to Figure 5). *Characterization of loss of v-ATPase subunit ATP6AP1 in the osteosarcoma cell line U2OS.*

(**A**) Immunoblot confirmation of CRISPR-mediated ATP6AP1 knockout.

(**B**) Growth phenotypes of WT and ATP6AP1^{KO} to assess ACT sensitivity. Data represents the mean ±SD of five independent experiments (dots), 2-way ANOVA.

(**C**) Routine respiration and complex IV activity measurements in WT and ATP6AP1^{KO} cells treated with ACT; dots are independent experiments. Data represents the mean ±SD of four independent experiments (dots), 2-way ANOVA.

(**D**) Mitochondrial fragmentation status assessed by TOM20-immunofluorescence; U2OS. Scale bar: 1 µm. Data represents the mean dots representing individual cells, 2-way ANOVA.

(**E**) $\Delta\Psi_m$ assessed via TMRM fluorescence intensity in WT and ATP6AP1^{KO}. Data are mean ±SD (8 fields of view from 4 biological replicates), 2-way ANOVA.

(**F**) Combined altered metabolic pathways in ATP6AP1^{KO} in HEK and U2OS; all pathways $p<0.05$; untargeted metabolomics.

All treatments performed in U2OS cells for 3 days if not indicated otherwise. Bars indicate mean of measurements with cells or independent experiments as dots. Statistical significance was performed using ANOVA, *p≤0.05, **p≤0.01. ***p≤0.001, ****p≤0.0001.

Supplementary Table 1

REAGENT or RESOURCE	SOURCE	IDENTIFIER
Antibodies		
MIC60	Proteintech	Cat# 10179-1-AP; RRID:AB_2127193
MIC27/APOOL	Sigma Aldrich	Cat# HPA000612; RRID:AB_1078594
YME1L1	Proteintech	Cat# 11510-1-AP; RRID:AB_2217459
LC3B	Novus Biologicals	Cat# NB600-1384; RRID:AB_669581
PARP	Cell signalling	Cat#9542; RRID:AB_2160739
LAMP2	Santa Cruz	Cat # sc-18822; RRID: AB_626858
LAMP1 (D2D11)	Cell Signalling	Cat#9091; RRID: AB_2687579)
BETA ACTIN	Proteintech	Cat# 66009-1-Ig, RRID:AB_2687938
MRPL44	Proteintech	Cat #16394-1-AP; RRID: AB_2146062
MRPS27	Proteintech	Cat #17280-1-AP; RRID: AB_2180510
OPA1	BD Biosciences	Cat #612606; RRID: AB_399888
STK11/LKB1	Santa Cruz	Cat # sc-374334; RRID: AB_10989381
v-ATPase ATP6AP1	Santa Cruz	Cat# sc-81886; RRID: AB_1119179
v-ATPase ATP6AP2	Sigma Aldrich	Cat# HPA003156; RRID: AB_1078245
v-ATPase B1/2	Santa Cruz	Cat # sc-55544; RRID: AB_831844
v-ATPase A1	Santa Cruz	Cat # sc-374475; RRID: AB_10987694
v-ATPase ATP6V1A	Proteintech	Cat # 17115-1-AP ; RRID: AB_2290195
SHMT2	ThermoFisher Scientific	Cat # PA5-32228; RRID: AB_2549701
HSP60	Abcam	Cat # ab46798; RRID: AB_881444
VDAC1/Porin	Abcam	Cat # ab14734; RRID: AB_443084
CTH / CSE	Proteintech	Cat #12217-1-AP; RRID: AB_2087497
TOM40	Santa Cruz	Cat #sc-11414; RRID: AB_793274
NDUFA9	Abcam	Cat # ab14713; RRID: AB_301431
MT-CO1	Abcam	Cat # ab14607; RRID: AB_2084810
MT-CO2	Abcam	Cat # ab79393; RRID: AB_1603751
SDHA	Abcam	Cat# ab14715; RRID: AB_301433
ATP5B	Abcam	Cat# ab14748; RRID: AB_301447
UQCRFS1	Abcam	Cat #ab14746; RRID: RRID:AB_301445
PHGDH	Proteintech	Cat#14719-1-AP; RRID: AB_2283938
TOM20	Santa Cruz	Cat#sc-17764; RRID: AB_628381
TOM20	Abcam	Cat# ab186735; RRID: AB_2889972
GLUT1	Abcam	Cat# ab14683; RRID: AB_301408
HIF1A	BD Transduction Labs	Cat#610958; RRID: AB_398271
FLAG M2	Sigma Aldrich	Cat # F1804, RRID: AB_262044
HRP-linked anti-mouse	Jackson ImmunoResearch	Cat# 115-035-146; RRID: AB_2307392
HRP-linked anti-rabbit	Jackson ImmunoResearch	Cat# 111-035-144; RRID: AB_2307391
Alexa488 anti-mouse	ThermoFisher Scientific	Cat# A-11001; RRID: 2534069
Alexa594 anti-rabbit	ThermoFisher Scientific	Cat# A-21207; RRID: AB_141637

Chemicals, Peptides and Recombinant Proteins		
DMEM	ThermoFisher Scientific	Cat # 41965-039
Sodium Pyruvate	Gibco	Cat#11360-039
Glutamax (L-Glutamine)	Gibco	Cat#35050-038
Fetal Bovine Serum	ThermoFisher Scientific	Cat#10500-064
Fetal Bovine Serum (dialysed)	ThermoFisher Scientific	Cat# A3382001
Non-essential amino acids	Gibco	Cat#11140-035
Uridine	Calbiochem	Cat#6680
Penicillin-Streptomycin	ThermoFisher Scientific	Cat#15140122
Puromycin Dihydrochloride	Sigma/Merck	Cat#P9620
Chloramphenicol-Water Soluble	Sigma/Merck	Cat#C0378-25G
Dulbecco`s 1xPBS	Corning	Cat#2140CV
RIPA buffer	ThermoFisher Scientific	Cat#89900
TWEEN-20	AMRESCO	Cat#0777-1L
Actinonin	Sigma/Merck	Cat#A6671-25MG
MitotrackerCMXROS	ThermoFisher Scientific	Cat#M7512
TMRM	ThermoFisher Scientific	Cat#T668
LysotrackerRed-DND99	Life technologies	Cat#L7528
pHrodo red/green AM	ThermoFisher Scientific	Cat#P35373
Hoechst 33342	Life technologies	Cat#H3570
Qiazol Lysis reagent	Qiagen	Cat#79306
Isopropanol	ThermoFisher Scientific	Cat# P/7490/PB17
DMSO	MP biomedicals	Cat#195066
Trypan Blue	Corning	Cat#25-900-CI
Deferoxamine	Sigma/Merck	Cat#D9533-1G
Ferric citrate	Sigma/Merck	Cat#F3388
Sodium citrate	Sigma/Merck	Cat#S-4641
Antimycin A	Sigma/Merck	Cat# A8674
TMPD	Sigma/Merck	Cat# T 3134
Ascorbate	Sigma/Merck	Cat# PHR1279-1G
Rotenone	Sigma/Merck	Cat# R8875
Malate	Sigma/Merck	Cat# M1000
Oligomycin	Sigma/Merck	Cat# O4876
Metformin	MecChem	Cat# HY-17471A
Sodium azide	Sigma/Merck	Cat# S2002
Glucose	Sigma/Merck	Cat# G7021
Galactose	Sigma/Merck	Cat# G0750
Concanamycin A	Santa Cruz	Cat# 81552-34-3
Balifomycin A1	Santa Cruz	Cat# 98813-13-9
FCCP	Sigma/Merck	Cat# C2920
N-Dodecyl-β-Maltoside	Sigma/Merck	Cat# D4641
D-Glucose-1,2-$^{13}C_2$	Sigma/Merck	Cat# 453188
Digitonin	Calbiochem	Cat# 300410
Mini Protease Inhibitor Cocktail	Roche	Cat# 11836153001
BCECF AM	ThermoFisher Scientific	Cat# B1170
Clarity Western ECL Substrate	Biorad	Cat# 1705060

Vectashield	VectorLabs	Cat# H-1000-10
Protein G Sepharose 4 Fasst Flow	Sigma/Merck	Cat# GE17-0618

Critical Commercial assays		
BioRad Stain-Free 4-20%	BioRad	Cat# 4568094
BioRad Stain-Free 7.5%	BioRad	Cat# 4568024
Native-PAGE Bis-Tris gels	Invitrogen	Cat# BN1001
SYBR Green supermix	BioRad	Cat# 1708880
Nucleospin DNA extraction kit	Macherey-Nagel	Cat# 740952.50
NebNext Ultra II Q5 Master mix	NewEnglandBiolabs	Cat# M0544S
QIAquick PCR purification kit	Qiagen	Cat# 28104
Trans-blot Transfer Turbo System	BioRad	Cat# 1704150
Nucleospin blood XL kit	Macherey-Nagel	Cat# 740950
iScript™ cDNA Synthesis Kit	Biorad	Cat# 1708891
Nucleospin blood XL kit	Macherey-Nagel	Cat# 740950
Intracellular pH calibration buffer kit	ThermoFischer	Cat# P35379
PierceTM BCA Protein Assay	ThermoFischer	Cat# A55865

Experimental models: Cell Lines		
HEK293T	ATCC	CRL-1573
U2OS	ATCC	HTB-96
HEK293T-Cas9	This study	
HEK293T-Cas9 pLJM1-FIRE-pHLy	This study	
HEK293-AT6AP1^{KO}	This study	
HEK293T-Cas9 pLJM1-FIRE-pHLy	This study	
U2OS-Cas9	This study	
U2OS-ATP6AP1^{KO}	This study	
HEK293-MRPL44^{KO}	Reference [1]	
HEK293-C6ORF203^{KO}	This study	
Control fibroblasts	Reference [2]	
MRPS14^{Pat}	Reference [2]	
MT-ATP6^{Pat}	Reference [3]	

Oligonucleotides (5`-3`)	
Universal gRNA_P5	TTGTGGAAAGGACGAAACACCG
Universal gRNA_P7	CCAATTCCCACTCCTTTCAAGACC
B2M_F_mtDNA	TGCTGCTCCATGTTTGATGTATCT
B2M_R_mtDNA	TCTCTGCTCCCCACCTCTAAGT
ATP8_F_mtDNA	ATGGCCCACCATAATTACCC
ATP8_R_mtDNA	CATTTTGGTTCTCAGGGTTTG
ND4_F_mtDNA	TCCTCCTATCCCTCAACCCC
ND4_R_mtDNA	CACAATCTGATGATGTTTTGGTTAAAC

Sequence gRNA (5`-3`) for specific KO	
Control_19 (non-target control #2)	GCGCGATAGCGCGAATATAC
Control_20 (non-target control #3)	TATGTGCGGCAAACCAAGCG
ATP6AP1 sg1	CCTGCGGCCGACACTCATGAAGG
ATP6AP1 sg2	ATTGAGGATTTCACAGCATATGG

Recombinant DNA		
lentiCRISPRv2	Addgene	Cat # 52961
Human Brunello CRISPR knockout pooled library	Addgene	Cat# 73178
pLJM1-FIRE-pHLy	Addgene	Cat#170775
ATP6AP1-FLAG	Genescript	Clone ID: OHu09985

Software and Algorithms		
Prism v.10.4.2	GraphPad	https://www.graphpad.com/
Illustrator CS6	Adobe	https://www.adobe.com/products/illustrator.html
Photoshop CS6	Adobe	https://helpx.adobe.com/fi/photoshop/
ImageJ / Fiji v.2.14.0	NIH	https://imagej.nih.gov/ij/
Incucyte2022B	Sartorius	https://downloads.essenbioscience.com/get/incucyte-2022b-rev1-gui
MAGeCK-VISPR	n/a	https://bitbucket.org/liulab/mageck-vispr
MetaboAnalyst 5.0	n/a	https://www.metaboanalyst.ca/
CellProfiler 4.2.5	n/a	https://cellprofiler.org/
Pymol v.2.4.1.	n/a	https://www.pymol.org/
ImageLab 6.1	Biorad	https://www.bio-rad.com/
R version 4.5.1	n/a	https://www.r-project.org/
Xcalibur 4.1.31.9	Thermo Scientific	https://www.thermofisher.com/
Perseus 1.6.15.0	n/a	https://maxquant.net/perseus
TraceFinder 4.1	Thermo Scientific	https://www.thermofisher.com/
Incucyte 2022B	Sartorius	https://www.sartorius.com/
Ingenuity Pathway Analysis	Qiagen	https://digitalinsights.qiagen.com

References

1. Hilander, T., Awadhpersad, R., Monteuuis, G., Broda, K.L., Pohjanpelto, M., Pyman, E., Singh, S.K., Nyman, T.A., Crevel, I., Taylor, R.W., et al. (2024). Supernumerary proteins of the human mitochondrial ribosomal small subunit are integral for assembly and translation. iScience *27*, 110185. https://doi.org/10.1016/j.isci.2024.110185.

2. Jackson, C.B., Huemer, M., Bolognini, R., Martin, F., Szinnai, G., Donner, B.C., Richter, U., Battersby, B.J., Nuoffer, J.-M., Suomalainen, A., et al. (2019). A variant in MRPS14 (uS14m) causes perinatal hypertrophic cardiomyopathy with neonatal lactic acidosis, growth retardation, dysmorphic features and neurological involvement. Hum. Mol. Genet. *28*, 639–649. https://doi.org/10.1093/hmg/ddy374.

3. Seneca, S., Abramowicz, M., Lissens, W., Muller, M.F., Vamos, E., and Meirleir, L. de (1996). A mitochondrial DNA microdeletion in a newborn girl with transient lactic acidosis. J. Inherit. Metab. Dis. *19*, 115–118. https://doi.org/10.1007/bf01799407.
