# Supplementary material for: Vacuolar-type H+-ATPase-mediated extra-organellar buffering resolves mitochondrial dysfunction
Source: Nat Commun. 2025 Dec 3;17:67. doi: 10.1038/s41467-025-66656-1 (PMC12769673; doi:10.1038/s41467-025-66656-1)
Supplement: Supplementary file 2 — Reporting Summary [file 41467_2025_66656_MOESM2_ESM.pdf]

nature portfolio

Corresponding author(s): Christopher B. Jackson

Last updated by author(s): 23/9/2025

Reporting Summary

Nature Portfolio wishes to improve the reproducibility of the work that we publish. This form provides structure for consistency and transparency in reporting. For further information on Nature Portfolio policies, see our Editorial Policies and the Editorial Policy Checklist.

Statistics

For all statistical analyses, confirm that the following items are present in the figure legend, table legend, main text, or Methods section.

n/a	Confirmed	
☐	☒	The exact sample size (*n*) for each experimental group/condition, given as a discrete number and unit of measurement
☐	☒	A statement on whether measurements were taken from distinct samples or whether the same sample was measured repeatedly
☐	☒	The statistical test(s) used AND whether they are one- or two-sided<br>*Only common tests should be described solely by name; describe more complex techniques in the Methods section.*
☐	☒	A description of all covariates tested
☐	☒	A description of any assumptions or corrections, such as tests of normality and adjustment for multiple comparisons
☐	☒	A full description of the statistical parameters including central tendency (e.g. means) or other basic estimates (e.g. regression coefficient) AND variation (e.g. standard deviation) or associated estimates of uncertainty (e.g. confidence intervals)
☐	☒	For null hypothesis testing, the test statistic (e.g. *F*, *t*, *r*) with confidence intervals, effect sizes, degrees of freedom and *P* value noted<br>*Give P values as exact values whenever suitable.*
☒	☐	For Bayesian analysis, information on the choice of priors and Markov chain Monte Carlo settings
☒	☐	For hierarchical and complex designs, identification of the appropriate level for tests and full reporting of outcomes
☒	☐	Estimates of effect sizes (e.g. Cohen's *d*, Pearson's *r*), indicating how they were calculated

Our web collection on statistics for biologists contains articles on many of the points above.

Software and code

Policy information about availability of computer code

Data collection	No unique software was used.
Data analysis	Data was analysed with GraphpadPrism10 (10.0.1), FIJI, CellProfiler4,and MAGeCK pipeline (Li et al., 2014).

For manuscripts utilizing custom algorithms or software that are central to the research but not yet described in published literature, software must be made available to editors and reviewers. We strongly encourage code deposition in a community repository (e.g. GitHub). See the Nature Portfolio guidelines for submitting code & software for further information.

Data

Policy information about availability of data

All manuscripts must include a data availability statement. This statement should provide the following information, where applicable:

- Accession codes, unique identifiers, or web links for publicly available datasets
- A description of any restrictions on data availability
- For clinical datasets or third party data, please ensure that the statement adheres to our policy

All data is available from the authors upon request.
There are no restrictions on data availability.

Research involving human participants, their data, or biological material

Policy information about studies with human participants or human data. See also policy information about sex, gender (identity/presentation), and sexual orientation and race, ethnicity and racism.

Reporting on sex and gender	The study did not involve human participants.
Reporting on race, ethnicity, or other socially relevant groupings	The study did not involve human participants.
Population characteristics	The study did not involve human participants.
Recruitment	The study did not involve human participants.
Ethics oversight	The study did not involve human participants.

Note that full information on the approval of the study protocol must also be provided in the manuscript.

Field-specific reporting

Please select the one below that is the best fit for your research. If you are not sure, read the appropriate sections before making your selection.

☒ Life sciences ☐ Behavioural & social sciences ☐ Ecological, evolutionary & environmental sciences

For a reference copy of the document with all sections, see nature.com/documents/nr-reporting-summary-flat.pdf

Life sciences study design

All studies must disclose on these points even when the disclosure is negative.

Sample size	Sample sizes were not predetermined based on pre-specified effect size, but were selected based on commonly adopted standards in the field taking into account previous experience and literature to ensure statistically meaningful comparisons and adequate statistical power. The numbers of samples analyzed are provided in the figure legends for all experiments.
Data exclusions	No samples were excluded.
Replication	For all quantified experiments, the number of independent replications is indicated in the figure legends. All graphs clearly indicate biological, technical or other replicates (e.g., individual cells).
Randomization	For all treatments, cultured cells of the required type were randomly allocated into control and treatment group without using a formalized randomization procedure.
Blinding	Investigators were generally not blinded as the experimental work flow required investigators to know the identity of the samples. However, data analysis occured in an unbiased manner by applying analysis routines from image analysis programs in an identical manner to all images of the different experimental groups.

Reporting for specific materials, systems and methods

We require information from authors about some types of materials, experimental systems and methods used in many studies. Here, indicate whether each material, system or method listed is relevant to your study. If you are not sure if a list item applies to your research, read the appropriate section before selecting a response.

Materials & experimental systems

n/a	Involved in the study	
☐	☒	Antibodies
☐	☒	Eukaryotic cell lines
☒	☐	Palaeontology and archaeology
☒	☐	Animals and other organisms
☒	☐	Clinical data
☒	☐	Dual use research of concern
☒	☐	Plants

Methods

n/a	Involved in the study	
☒	☐	ChIP-seq
☒	☐	Flow cytometry
☒	☐	MRI-based neuroimaging

Antibodies

Antibodies used	Primary antibodies<br>MIC60 Proteintech Cat# 10179-1-AP; RRID:AB_2127193<br>MIC27/APOOL Sigma Cat# HPA000612; RRID:AB_1078594<br>YME1L1 Proteintech Cat# 11510-1-AP; RRID:AB_2217459<br>LC3B Novus Biologicals Cat# NB600-1384; RRID:AB_669581<br>p62 abcam Cat# ab56416; RRID:AB_945626<br>OMA1 Proteintech Cat#17116-1-AP; RRID:AB_2299053<br>LAMP1 (D2D11) Cell Signalling Cat#9091; RRID: AB_2687579)<br>LAMP2 Santa Cruz Cat # sc-18822; RRID: AB_626858<br>MRPL44 Proteintech Cat #16394-1-AP; RRID: AB_2146062<br>MRPS27 Proteintech Cat #17280-1-AP; RRID: AB_2180510<br>OPA1 BD Bioscences Cat #612606; RRID: AB_399888<br>v-ATPase ATP6AP1 Satna Cruz Cat# sc-81886; RRID: AB_1119179<br>v-ATPase ATP6AP2 Sigma Aldrich Cat# HPA003156; RRID: AB_1078245<br>v-ATPase B1/2 Santa Cruz Cat # sc-55544; RRID: AB_831844<br>V-ATPase A1 Santa Cruz Cat # sc-374475; RRID: AB_10987694<br>SHMT2 ThermoFisher Scientific Cat # PA5-32228; RRID: AB_2549701<br>HSP60 Abcam Cat # ab46798; RRID: AB_881444<br>VDAC1/Porin Abcam Cat # ab14734; RRID: AB_443084<br>CTH / CSE Proteintech Cat #12217-1-AP; RRID: AB_2087497<br>TOM40 Santa Cruz Cat #sc-11414; RRID: AB_793274<br>NDUFA9 Abcam Cat # ab14713; RRID: AB_301431<br>MT-CO1 Abcam Cat # ab14607; RRID: AB_2084810<br>MT-CO2 Abcam Cat # ab79393; RRID: AB_1603751<br>SDHA Abcam Cat# ab14715; RRID: AB_301433<br>ATP5B Abcam Cat# ab14748; RRID: AB_301447<br>UQCRFS1 Abcam Cat #ab14746; RRID: RRID:AB_301445<br>VINCULIN Abcam Cat# ab129002; RRID: AB_11144129<br>PHGDH Proteintech Cat#14719-1-AP; RRID: AB_2283938<br>TOM20 Santa Cruz Cat#sc-17764; RRID: AB_628381<br>TOM20 Abcam Cat# ab186735; RRID: AB_2889972<br>FLAG M2 Sigma Cat # F1804, RRID: AB_262044<br><br>Secondary antibodies<br>HRP-linked anti-mouse Jackson ImmunoResearch Cat# 115-035-146; RRID: AB_2307392<br>HRP-linked anti-rabbit Jackson ImmunoResearch Cat# 111-035-144; RRID: AB_2307391<br>Alexa488 anti-mouse ThermoFisher Scientific Cat# A-11001; RRID: 2534069<br>Alexa594 anti-rabbit ThermoFisher Scientific Cat# A-21207; RRID: AB_141637
Validation	Antibodies against ATP6AP1, MRPL44 were validated by gene knockout (this study) and FLAG by overexpression.<br>Antibodies against OXPHOS complexes have been validated extensively for immunoblotting in denaturing and native PAGE analyses (Jackson et al., 2019). All other antibodies used in this study have been validated before and unanimously confirmed convergent mass spectrometry results in this study.

Eukaryotic cell lines

Policy information about cell lines and Sex and Gender in Research

Cell line source(s)	HEK293(#CRL-1573) and U2OS (#HTB-96) cell lines were originally obtained from ATCC. Fibroblasts cell lines were obtained as indicated in respective publications with the respective gender indicated.
Authentication	Cell lines from ATCC are regularly authenticated by STR profiling.
Mycoplasma contamination	All cell lines were regularly tested for mycoplasma contamination.
Commonly misidentified lines<br>(See ICLAC register)	There were no commonly misidentified cell lines used in this study.

Plants

Seed stocks	n/a
Novel plant genotypes	n/a
Authentication	n/a
